# A Simple Sperm DNA Toroid Integrity Test and Risk of Miscarriage

**DOI:** 10.1155/2015/780983

**Published:** 2015-01-15

**Authors:** Philip J. Chan, Eliza M. Orzylowska, Johannah U. Corselli, John D. Jacobson, Albert K. Wei

**Affiliations:** Department of Gynecology and Obstetrics, Loma Linda University School of Medicine, Loma Linda, CA 92354, USA

## Abstract

Current methods of analyzing sperm chromatin competency overlook the inner sperm compartment which is inaccessible to probes and reagents. By breaking the molecular protamine disulfide bridges, the DNA toroids are exposed to integrity analysis. The aim was to develop a simple nuclear toroid test and determine its association with fertilization, pregnancy, and miscarriage. The approach involved treating washed sperm remaining after ICSI procedures (*N* = 35 cases) with acidified Triton X-100 and dithiothreitol (DTT) before Diff-Quik staining. Percentages of sperm with normal chromatin indicated by light-colored nuclei were assessed. The toroid integrity test showed more sperm with normal chromatin in the pregnant group (73.6 ± 1.7%, mean ± SEM) when compared with the miscarriage (51.2 ± 6.6%) or nonpregnant groups (60.9 ± 4.8%). Furthermore, the toroid results were correlated with ICSI fertilization (*R* = 0.32, *P* = 0.04) and pregnancy outcome (pregnant cases 73.6 ± 1.7% versus nonpregnant 58.0 ± 3.9%, *P* = 0.001). ROC calculated cut-off was >70.0% for normal toroid integrity (sensitivity 0.98, specificity 0.33, and diagnostic accuracy 78.3%). An association between normal sperm toroid integrity and miscarriage was evident when the staining procedure included acidified detergent DTT pretreatment.

## 1. Introduction

During spermatogenesis, human sperm DNA is remodeled into highly compacted doughnut-shaped protamine “toroids” or coils of condensed DNA [1, 2] and held together by disulfide bonds formed by the oxidation of sulfhydryl groups on the protamines. About 10–15% of somatic histones are retained by the sperm nucleus which take part in decompression after fertilization to expose reading frames for protein synthesis at late stage embryonic development [3, 4]. The sperm DNA nuclear toroids are attached to the nuclear matrix (scaffolding) at matrix attachment regions (MARs) at intervals of about 50 kb throughout the genome [4–7]. Damage to sperm DNA in these regions has been linked to decreased fertilization, failed implantation, miscarriage, and birth defects and these findings have been reviewed [8, 9].

Several test assays to assess DNA damage or fragmentation have been reported including the sperm chromatin structure assay (SCSA) [10], sperm chromatin dispersion (SCD) [11], single-cell gel electrophoresis or comet assay [12], annexin-V assay [13], terminal deoxynucleotidyl transferase-mediated deoxyuridine triphosphate biotin nick-end labeling assay (TUNEL) [14], and the Acridine orange test (AOT) [15, 16]. The characteristics of these assays have been reviewed by numerous groups [17–21]. However, these assays are complicated and time-consuming, require specialized equipment, and focused on DNA damage. Furthermore, some of the assays, such as SCSA, AOT, and TUNEL, provide limited information due to the inaccessibility of the inner sections of the compacted nucleus by the assay reagents. This difficulty has been elegantly demonstrated by researchers using combined detergent and reducing chemical agents that yield different results when compared with well-known sperm DNA assay methodology [7, 22, 23].

The problem of access to the inner sperm compartment was central in opening a gateway to the development of the present simple sperm toroid integrity (STI) test for sperm chromatin integrity. The premise here was that, by using combined reagents to deliberately break the intra- and intermolecular protamine disulfide bridges [22–24], increased accessibility of the freed DNA toroids to analytical reagents would be accomplished. Consequently, abnormal sperm toroid configurations due to DNA fragmentation and/or abnormal chromatin arrangement would be detected as enhanced staining of DNA dyes resulting in extra dark stained nuclei. However, a combination of detergent and reducing agents may possibly damage chromosomes due to membrane-released endonucleases [7, 25]. To avoid this damage, acidified reagents were used, incubation time reduced to 5 minutes, and the reagents were prepared in calcium and magnesium-free saline. The objectives of the study were to use the simple STI test procedure (a) to ascertain the correlation between sperm normal toroid integrity and oocyte fertilization by ICSI, (b) to study the relationship between sperm normal toroid integrity and pregnancy outcome, and (c) to determine the association between sperm normal toroid integrity and miscarriage rate.

## 2. Materials and Methods

### 2.1. Patients

The project was reviewed and approved by the Institutional Review Board. Semen was obtained from the male partners (age 36.5 ± 1.0 years, mean ± SEM) of 35 female patients (age 33.8 ±0.8 years) undergoing in vitro fertilization (IVF) treatment with the intracytoplasmic sperm injection (ICSI) procedure (Table 1). Female patients were selected based on adequacy of sperm cells remaining after the ICSI procedure. Exclusion criteria were patients undergoing banked embryo cycles, donor sperm cycles, or conventional IVF cycles. Primary diagnoses of the patients were 15 diminished ovarian reserve, 8 tubal, 5 male factor, 3 endometriosis, 2 unexplained, and 2 polycystic ovarian syndrome cases. The IVF and ICSI procedures were based on established protocols [26]. Briefly, patients scheduled for the IVF procedure at the Center for Fertility and In Vitro Fertilization were given routine examinations. The ovulation induction treatment of each female patient was initiated with 0.5 mg leuprolide acetate for pituitary down regulation beginning on day 21 of the previous cycle and ending on the last day of stimulation. Ovarian follicular growth was stimulated using recombinant follicle stimulating hormone (an average of 4 ampules in split doses daily, 75 I.U. per ampule) for 9 to 12 days. Ovulation was triggered by administering 10,000 units of human chorionic gonadotropin on the day when there was a group of follicles with the largest follicle at over 18 mm diameter and serum estradiol concentration reflective of the follicles (200 pg/mL per follicle).

On the day of oocyte retrieval, sperm of male partners were washed for the ICSI procedure using the discontinuous 2-layer 90 : 45% colloid (Isolate; Irvine Scientific, Santa Ana, CA) gradient centrifugation procedure [27]. The processed sperm were resuspended in HEPES-based HTF medium (synthetic human tubal fluid, Irvine Scientific, Santa Ana, CA) supplemented with 5% human serum albumin at a concentration of 10 million/mL with at least 90% total motility. Motile sperm were selected by swimming out and used in the ICSI procedure. For each patient, the remaining portion of sperm cells not used in the ICSI procedure was divided into the treatment and control groups and processed as described below.

The medium used for all embryo cultures was G1.3 Plus medium (Vitrolife Fertility Systems, Englewood, CO). After 18–24 hours, the ICSI-treated oocytes were examined. An oocyte was classified as fertilized when 2 pronuclei were present in the ooplasm. Day 5 embryos at the blastocysts stage were transcervically returned to the uterine lumen of the patients and monitored for evidence of pregnancy. In this study, the miscarriage group was comprised of 5 cases of miscarriage (pregnancy loss, spontaneous abortion) following ultrasound confirmation of a gestational sac plus a single case of biochemical pregnancy. Altogether in the group of 35 patients, there were 15 pregnant cases with live births, 6 pregnant cases with pregnancy loss, and 14 nonpregnant cases. The possibility of very early miscarriages in the nonpregnant patients could not be ascertained due to the inadequacy of existing test methods.

### 2.2. The Simple Sperm Toroid Integrity (STI) Test Procedure

The treatment for the STI test procedure required only 2 main reagents, Triton X-100 (Sigma-Aldrich Co., St. Louis, MO) and dithiothreitol (DTT, IBI Scientific, Peosta, IA). In addition, a standard sperm staining kit that stained DNA (Diff-Quik stain kit, catalog number B4132-1A, Siemens Healthcare Diagnostics, Newark, DE) was required. Alternative stains including the toluidine blue, Hemacolor, and Giemsa stain kits have been reported but were not used here [28–30].

The STI test procedure began by taking washed sperm from each patient and dividing them into 2 groups: treated (STI) and untreated (control) groups. The treatment for the STI group was as follows. An aliquot (0.1 mL) of washed sperm (concentration 100,000 sperm/mL) was pipetted into a microcentrifuge tube immediately followed by an equal volume (0.1 mL) of a solution of acid-detergent Triton X-100 (a mixture of 0.1% Triton X-100, 0.15 M NaCl, and 0.08 M HCl, pH 1.2) [31]. The purpose of the acidified Triton X-100 was to permeabilize the sperm membrane. This solution was stable for a week under refrigerated conditions.

Immediately following the addition of the acidified Triton X-100 solution, 0.1 mL of a DTT stock solution was added into the mixture in the same microcentrifuge tube (final concentration of 2 mM DTT) and incubated at 37°C for 5 minutes. The short 5-minute incubation time was important as longer periods would result in possible chromosomal damage from membrane derived endogenous nucleases [7, 25]. The purpose of the reducing agent DTT was to open up the chromatin structure by breaking the disulphide bridges between protamine molecules [22–24, 28]. The DTT reagent was stored frozen in aliquots of 0.5 mL in microcentrifuge vials and thawed just before use in the mixture. An alternative to DTT, such as Tris (2-carboxyethyl) phosphine hydrochloride (TCEP), prepared at the same concentration could also be used as a more pH-stable substitute. The result (presented in the next section) was comparable for either DTT or TCEP for the short incubation period.

After 5 minutes of incubation, the treated sperm were smeared on glass slides and air-dried. For the control group, smears of untreated sperm were made on separate slides. For convenience, dried sperm slides could be fixed in 100% methanol for 10 seconds, air-dried, and stored in a dark location until the staining procedure. The staining procedure was carried out on both STI treatment and control slides. Basically, the Diff-Quik stain procedure [30] was used with a minor modification. After rinsing off the final stain (methylene-blue mixture), the sperm slides were immersed under water for a minute to remove the excess stain. The slides were air-dried and sperm status was evaluated under oil immersion light microscopy (×1000) based on previously published modified Diff-Quik protocol for sperm morphology and chromatin status [30].

**Figure 1:**
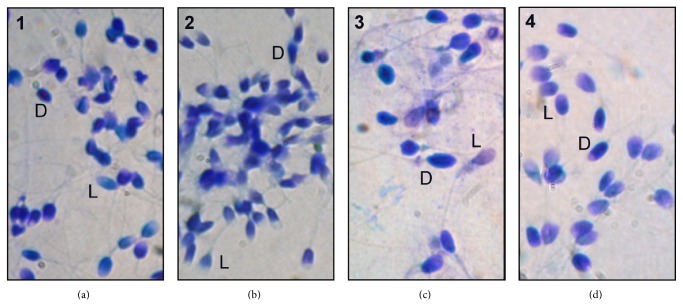
A simple sperm toroid integrity (STI) test of sperm quality in the male partner of patients undergoing IVF with ICSI treatment. Sperm were pretreated with acidified detergent dithiothreitol (DTT) and stained with the Diff-Quik stain kit followed by immersion in water for one minute. Sperm with abnormal chromatin had dark-colored nuclei (type D, only 1 sperm labeled per panel as an example) in contrast with light-colored normal chromatin nuclei (type L). Panels 1 to 3 were from different patients with miscarriage, while Panel 4 was from a patient with live-birth outcome.

Sperm cell nuclei that stained either intensely dark blue or dark violet were considered abnormal (type D, Figure 1). Sperm with slightly dark shade-colored or light-colored nuclei were considered normal (type L, Figure 1). Acephalic sperm or swollen decondensed sperm were not analyzed. Sperm tail staining dark blue indicated overstaining and required further soaking in water for a longer period. The percentages of normal chromatin sperm characterized by light-colored sperm nuclei (type L) were determined in the STI test treatment and control slides. Consistent with the practice of reporting normal sperm parameters such as normal morphology or viability, the percentage of sperm with normal toroid integrity was reported instead of the percentage of sperm with abnormal nuclear toroids. A total of 200 sperm cells were analyzed for each ICSI patient. The results were recorded and correlation analyses with the strict morphology, fertilization, and pregnancy outcome parameters were performed.

### 2.3. Sperm Strict Normal Morphology Analyses

Each Diff-Quik stained sperm slide was analyzed using oil immersion (1000x) bright field light microscopy for normal morphology based on the Tygerberg strict criteria method [31]. A sperm was classified as normal when the head was oval with the acrosome occupying 40–70% of the head, absence of midpiece and tail defects, and absent or small cytoplasmic droplets with the appropriate head dimensions. The percentage of sperm with strict normal morphology was calculated by dividing the number of sperm with normal morphology over the total number of sperm analyzed and multiplied by 100. A specimen was classified in the normal sperm morphology category when the percentage of strict normal morphology was over 4%. At least 200 sperm were analyzed from different locations on the sperm slide by a single technician.

### 2.4. Statistical Analysis

The results of the sperm parameter measurements were expressed as mean ± S.E.M. (standard error of the mean). The significance of *R*, correlation coefficient, was tested using linear regression statistics (Epistat Services, Richardson, TX). Basically, linear regression involved calculating sum of squares for the data points of each parameter followed by calculating the coefficient of determination, *R*-squared. The correlation coefficient, *R*, was derived from the *R*-squared value and significantly determined using *t*-tables computed in the Epistat software. Means were checked for equality of variance and tested using the Students' *t*-test two-tailed statistics (http://www.OpenEpi.com, Open Source Epidemiologic Statistics for Public Health, Atlanta, GA). Categorical data were analyzed using chi-square test statistics. A value of *P* < 0.05 was considered significant. Receiver operator characteristic (ROC) curve analysis was used to obtain the cut-off value and sensitivity and specificity of the STI test procedure based on different percentages of sperm with normal toroid integrity in relation to the incidence of miscarriage.

## 3. Results

**Figure 2:**
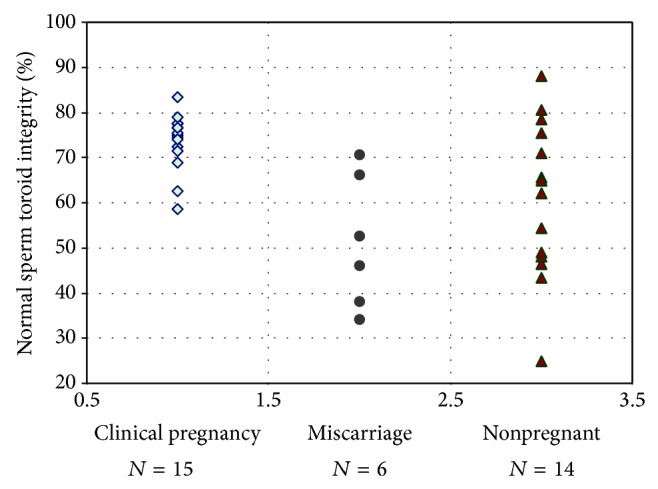
Pregnancy outcomes and the percentages of sperm with normal toroid assessed using the simple sperm toroid integrity (STI) test procedure in the male partner of patients undergoing IVF with ICSI treatment. Mean percentage of cases with clinical pregnancy and live-birth outcome (73.6 ± 1.7%) was higher (*P* < 0.05) when compared with either miscarriage cases (51.2 ± 6.6%) or nonpregnant cases (60.9 ± 4.8%).

In the STI test treatment group, the mean percentage of sperm with normal toroid integrity (orthochromatic or lightly-stained nuclei) was significantly (*P* < 0.05) higher (Figure 2) in the male partner of pregnant patients with live-birth outcome (73.6 ± 1.7%) when compared with either pregnant patients that miscarried (51.2 ± 6.6%) or nonpregnant patients (60.9 ± 4.8%). There was no difference in the percentage of normal sperm toroid status between the miscarriage and nonpregnant groups. The STI testing procedure showed that the percentages of sperm with normal toroid integrity in all 6 miscarriage cases were below 71.0% (Figure 2). There were no pregnancies when the percentage of sperm with normal toroid integrity was below 59%. The reference value for normal STI test was determined to be >70.0% with sensitivity of 0.98, specificity of 0.33, diagnostic accuracy of 78.3%, precision of 66.7%, and an area under the ROC curve of 0.71. The intra-assay and interassay coefficients of variation for STI test analyses were 6 and 4%, respectively. The statistical power calculated by normal approximation method was 94.9% for the pregnant group versus the miscarriage group and 73.3% for the pregnant group versus the nonpregnant group.

In contrast to the STI test group, in the control group, wherein the acidified detergent disulfide reduction treatment of sperm was not used, there were no differences observed. Note that the staining procedure used in the control group was similar to the previously reported chromatin testing methodology [30]. In the control group here, the percentages of sperm with normal toroid integrity in the pregnant patients with a positive outcome group (74.4 ± 3.2%), miscarriage group (68.3 ± 7.3%), and nonpregnant group (71.4 ± 3.6%) were similar. In terms of the percentages of sperm with strict normal morphology, there were no differences in the 3 groups (7.0 ± 1.4, 6.8 ± 2.6, and 8.6 ± 1.3%; pregnant, miscarriage, and nonpregnant groups, resp.).

**Figure 3:**
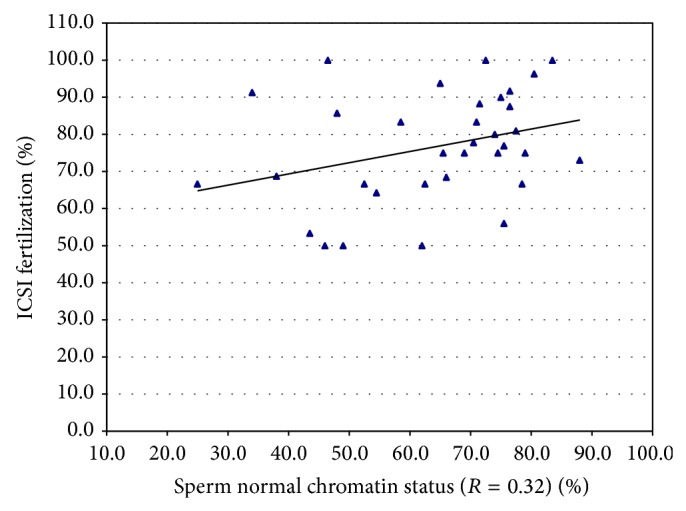
Scatter plot diagram showing the correlation (*P* < 0.04) between the percentage of sperm with normal sperm toroid chromatin status and percentage of oocyte fertilization after the ICSI procedure.

In the STI treatment group, correlation regression analysis showed an association (Figure 3) between normal sperm toroid integrity and ICSI fertilization (*R* = 0.32, *P* < 0.04) and pregnancy outcome. In the control group, sperm toroid integrity was correlated with ICSI fertilization (*R* = 0.71, *P* < 0.001) but not with pregnancy outcome. Statistical analysis showed no significant difference between sperm strict normal morphology and ICSI fertilization (*R* = 0.27, *P* > 0.06).

The mean percentage of sperm with normal toroid integrity was associated with live-birth pregnancy outcome (15 cases, 73.6 ± 1.7%, versus 20 nonpregnant cases, 58.0 ± 3.9%; *P* = 0.001). However, there was no difference in the untreated control sperm group (pregnant cases 74.4 ± 3.2% versus nonpregnant cases 70.5 ± 3.1%). In terms of an association between strict normal morphology and pregnancy outcome, no difference was observed (pregnant cases 7.0 ± 1.4% versus nonpregnant cases 8.1 ± 1.1%).

In the present group of 35 males with mean age 36.5 ± 1.0 years (median 36.0 years old), there were only 6 males over 40 years old. Linear regression analysis did not show a significant correlation between STI results and male age. TCEP, a substitute for DTT, known to possess greater stability under different pH conditions was tested in a separate STI test preparation procedure. The TCEP data based on a single washed sperm specimen repeated 10 times showed similar results for the mean percentage of normal toroid integrity (50.0 ± 1.2 versus 49.0 ± 1.3%, TCEP versus DTT, resp.). Several parameters that impacted fertilization and pregnancy outcome included maternal age, the number of high-quality embryos at the time of transfer, and the number of embryos transferred. There were no significant differences in these basic parameters of the patients for this study (Table 1).

## 4. Discussion

The present study demonstrated a positive relationship between miscarriage and abnormal sperm toroid integrity determined after STI testing (acidified detergent DTT) of washed sperm. The treatment was designed to challenge the sperm toroid chromatin integrity by deliberately breaking the intra- and intermolecular protamine disulfide bridges and solubilizing the nuclear matrix [22–24] to reveal the true status of the compacted DNA toroids. In this manner, a sperm nucleus with cumulative abnormalities resulting from damaged DNA, abnormal chromatin structures, and/or unstable toroids could be detected by the intense dark staining. Previous studies using DTT-treated sperm and toluidine blue staining reported increased metachromatic staining in infertile patients [28, 29]. It was postulated that the dark staining of abnormal sperm nuclei was due to increased accessibility to DNA phosphates in the abnormal crosslinked nucleus or in exposed nuclear foci sites in damaged DNA [28–30]. The accumulation of nuclear foci is a normal chromatin response to DNA damage that functions to protect genomic integrity [32]. The origins of DNA damage [33], abnormal chromatin [34, 35], and toroid dynamics [36–38] have been extensively reviewed.

In contrast to the STI test results, analyses of matched untreated sperm did not show a relationship between sperm DNA status and the incidence of miscarriage. This suggested that the combined acidified detergent DTT treatment to remove part of the sperm nuclear scaffolding was essential for actual determination of nuclear integrity [24]. The treatment also resulted in increased access into the nucleus for the polychromatic stains which led to improved visual detection of toroid abnormalities in sperm chromatin.

The incidence of miscarriage or pregnancy loss is reported to be about 15% of clinical pregnancies in the fertile population and varies according to age [20, 39, 40]. Possible causes of miscarriage range from thyroid autoimmunity, abnormal HLA-G expression to disrupted paternal genome [40]. The results here confirmed that, in the group of patients with identified miscarriage or pregnancy loss, the paternal genome component played an important role in the maintenance of pregnancy [4]. This finding was consistent with several studies reporting an association between loss of sperm nuclear integrity (DNA damage or fragmentation, abnormal chromatin remodeling, unbalanced histones-protamines ratio, abnormal matrix attachment region, MAR, or nuclear matrix scaffolding) and miscarriage [20, 40–45]. Additionally, the findings in the present study confirmed the results of other researchers that sperm nuclear integrity was predictive of pregnancy outcome [9, 46, 47].

Interestingly, the percentage of fertilized oocytes by ICSI was positively correlated with the normal sperm toroid integrity determined by both the STI test and standard Diff-Quik staining. This finding was in contrast with the majority of studies that did not indicate an association between fertilization rates and sperm DNA fragmentation [48, 49]. A possible explanation was that the STI test was not just another DNA fragmentation test but was designed to assess the cumulative sperm chromatin integrity. A previous report by Sousa and colleagues [30] did not find a correlation between fertilization and chromatin status. However, their study was based on sperm data from combined conventional IVF and ICSI cycles, while only ICSI cycles were analyzed here.

An advantage of the STI test described here was its simplicity which consisted of an easy 5-minute preliminary treatment of sperm with acidified detergent DTT. The treatment could be easily added to the routine Diff-Quik procedure for sperm morphology assessment in the laboratory. Expensive assay kits and equipment were not needed and only a light microscope with oil immersion capabilities was used in the STI test procedure. Shortcomings of the STI test included lack of capacity to discern severity or levels of sperm DNA damage, differentiating between sperm chromatin fragmentation (SCF) and sperm DNA fragmentation (SDF), and detecting epigenetic imprinting abnormalities and the requirement of fresh reagents, particularly DTT [50].

In summary, the STI test procedure was simple, was relatively rapid to perform, and required only a light microscope. Updating the routine laboratory semen analysis to include sperm DNA toroid integrity testing is essential in the clinical management of male factor infertility, as well as confirming the paternal contribution in miscarriages. More studies are still needed to optimize the STI test and improve its diagnostic accuracy for routine use in the ART IVF laboratories.

## Figures and Tables

**Table 1:** Basic parameters for the 35 IVF cycles with the ICSI procedure. Values are presented as mean ± SEM

Parameter	Pregnant live birth	Pregnant miscarriage	Nonpregnant
Number of patients (*N*)	15	6	14
Female age (years)	32.1 ± 1.0	34.5 ± 2.4	35.1 ± 1.2
Number of quality embryos	10.1 ± 1.1	11.8 ± 2.8	9.5 ± 1.8
Number of embryos transferred	1.9 ± 0.1	2.7 ± 0.4	2.4 ± 0.2
Male age (years)	34.4 ± 0.9	38.8 ± 3.2	37.9 ± 1.9
Semen volume (mL)	3.2 ± 0.4	2.7 ± 0.5	3.2 ± 0.3
Washed sperm concentration (10^6^/mL)	27.5 ± 5.0	34.0 ± 15.1	31.1 ± 5.9
Washed sperm total motility (%)	75.1 ± 4.5	76.3 ± 5.7	73.9 ± 4.7
